# Refinement and Validation of the Minimal Information Data-Modelling (MID) Method for Bridge Management

**DOI:** 10.3390/s24123879

**Published:** 2024-06-15

**Authors:** Connor O’Higgins, David Hester, Patrick McGetrick, Wai Kei Ao, Elizabeth J. Cross

**Affiliations:** 1 School of Natural and Built Environment, Queen’s University Belfast, University Rd., Belfast BT7 1NN, UK; c.ohiggins@qub.ac.uk; 2 School of Engineering, National University of Ireland Galway, University Rd., H91 TK33 Galway, Ireland; patrick.mcgetrick@universityofgalway.ie; 3 Department of Civil and Environmental Engineering, The Hong Kong Polytechnic University, Hong Kong; waikei.ao@polyu.edu.hk; 4 Department of Mechanical Engineering, University of Sheffield, Mappin Street, Sheffield S1 3JD, UK; e.j.cross@sheffield.ac.uk

**Keywords:** Structural Health Monitoring, data modelling, environmental effects, low cost, long-term bridge monitoring, regression

## Abstract

Various approaches have been proposed for bridge structural health monitoring. One of the earliest approaches proposed was tracking a bridge’s natural frequency over time to look for abnormal shifts in frequency that might indicate a change in stiffness. However, bridge frequencies change naturally as the structure’s temperature changes. Data models can be used to overcome this problem by predicting normal changes to a structure’s natural frequency and comparing it to the historical normal behaviour of the bridge and, therefore, identifying abnormal behaviour. Most of the proposed data modelling work has been from long-span bridges where you generally have large datasets to work with. A more limited body of research has been conducted where there is a sparse amount of data, but even this has only been demonstrated on single bridges. Therefore, the novelty of this work is that it expands on previous work using sparse instrumentation across a network of bridges. The data collected from four in-operation bridges were used to validate data models and test the capabilities of the data models across a range of bridge types/sizes. The MID approach was found to be able to detect an average frequency shift of 0.021 Hz across all of the data models. The significance of this demonstration across different bridge types is the practical utility of these data models to be used across entire bridge networks, enabling accurate and informed decision making in bridge maintenance and management.

## 1. Introduction

Bridges are critical components of transportation infrastructure and require a significant number of inspections and maintenance activities. Structural Health Monitoring (SHM) is one method that has been explored in recent times to aid the decision making undertaken by bridge managers. However, the widespread adoption of SHM for use in bridge management has not occurred. One reason for this is the need for a large number of sensors, which increases the costs associated with SHM systems.

In the field of bridge SHM, vibration-based monitoring is commonplace, and most of these studies have been conducted on long-span bridges. Many studies have been carried out on bridges that are over 150 m long [1,2,3,4,5,6,7], and a large number of sensors are typically installed for the Structural Health Monitoring of civil structures. For instance, Ref. [3] installed 232 sensors on a 1.1 km bridge, Ref. [4] installed more than 600 sensors on a tower, and [5] installed 114 sensors on a 168 m bridge. Although there have been some monitoring efforts on short- and medium-span bridges [8,9,10,11], they still follow the convention of installing a large number of sensors, with the sensors in these studies ranging from 40 to over 200. However, it is impractical to use large numbers of sensors across an entire bridge network due to logistical and financial limitations. Some studies have investigated the use of minimal, low-cost sensors for SHM, but these studies are often restricted to laboratory settings [12] or carried out on a short-term basis (e.g., 30 min [13] or 30 days [14]).

While existing methods of vibration-based bridge SHM may offer insights into bridge health, they often rely on resource-intensive data collection processes, as shown above. This approach may not be practical for many bridges, particularly the most common highway bridges. Moreover, the scarcity of long-term frequency data for short- and medium-span bridges poses challenges in validating SHM methods.

The majority of bridges in use today are short- and medium-span bridges. If SHM technology were widely adopted for these bridge types, the benefits could be significant. For SHM to be suitable for use across a range of short- and medium-span bridges, it will likely need to have the following properties:

1. Low cost: The cost of the system should be low so that it is feasible to install the system across a network of similar bridges, such as typical highway bridges.
2. Easy to install: The installation process should be straightforward enough so that it can be carried out by existing staff and minimise costly infrastructure disruptions.
3. Can provide useful information: The system should be able to determine when a bridge is behaving differently from the expected or previously measured behaviour.

To achieve these characteristics, the authors in [15] proposed to use a single MEMS accelerometer in conjunction with a sparse data modelling approach. The study demonstrated the concept on a single-span half-through girder bridge with data spanning approximately two years and identified that they could potentially identify a local stiffness loss of 23.6% and global damages of 1.7%. While the work of [15] was useful in demonstrating the concept, it is unknown if local and global stiffness loss detection capability were specific to the studied bridge or if the method would remain performant across a range of bridge types. Furthermore, Ref. [15] provides no information on whether the MID process is scalable. Specifically, if the user wished to apply to bridges on a network, a number of important questions arise that were not examined in [15]. For example, how repeatable and automated the method could be when monitoring different bridges.

This paper’s specific contributions can be summarised in 1 and 2 below.

1. Utilising data from four bridges to validate and refine the accuracy and reliability of MID data models for a range of different bridge types.
2. Developing tools and processes to ensure that MID is scalable when applied to multiple bridges.

The refinement process (contribution 1) aims to improve the accuracy of predictions regarding bridges’ dynamic behaviour while maintaining the method’s efficiency and practicality. With this increased accuracy, it is hoped that a smaller abnormal change in the natural frequencies of the bridges may be detected. After refining and validating the MID process, a standardised method was developed to test each MID data model to identify the detectable level of natural frequency shift.

The results of this study showed a high level of accuracy in predicting the bridges’ future dynamic behaviour. The average detectable frequency shift of all 15 data models was 0.021 Hz. This accuracy was achieved using an easy-to-use monitoring system costing only GBP 300.

## 2. Concept Overview

The goal of the MID methodology is to track and detect abnormal changes in a given natural frequency of a bridge. Using the data model to predict future behaviour current measurements of natural frequency can be compared to the predicted natural frequencies to allow any anomalous change in frequency to be identified. Damage to the bridge will cause a change in the natural frequency, and the smaller the frequency shift that can be detected, the more sensitive to changing natural frequency the data model will be.

**Figure 1:**
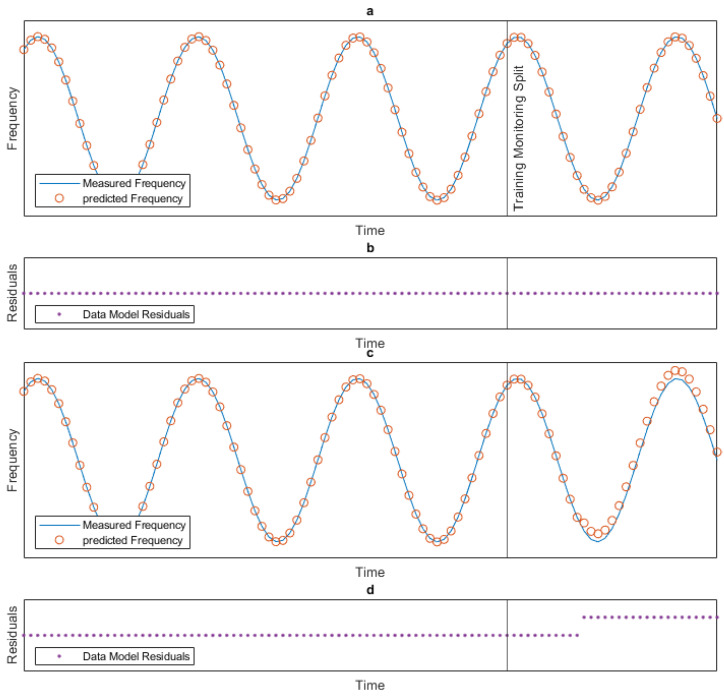
Concept schematic. Data model (**a**) with no abnormal behaviour. (**b**) residuals with no abnormal behaviour. (**c**) with abnormal behaviour. (**d**) residuals with abnormal behaviour.

A schematic of this concept is shown in Figure 1. Figure 1a shows the results of a theoretical data model. The blue line shows the actual natural frequency of the structure, which we can measure with instrumentation. The orange dots show the predicted natural frequency resulting from our trained data model. The black vertical line in Figure 1a–d denotes where the data were split so that data to the left of this line can be used to train the model, and data to the right denotes the data that are being monitored to detect abnormal changes in the frequency. In Figure 1a, there is no anomalous behaviour, so the blue line and orange dots perfectly align. This can be seen more clearly in Figure 1b, which shows the residuals (purple dots) of the data model, which are the predicted frequencies minus the measured frequencies. As the data model perfectly predicts the natural frequencies, the residuals of the data model are zero. Figure 1c,d show what happens when the natural frequency changes abnormally during the monitoring period. In the data model results shown in Figure 1c, it can be seen that the measured frequencies and the predicted frequencies diverge during the monitoring period; however, it is clearer in the residual plot shown in Figure 1d, with the point of abnormal behaviour being evident where the residual jumps up. This is the concept that will be utilised in this paper to detect abnormal behaviour in the monitored structures.

MID employs various data modelling techniques suitable for the kind of bridge data obtained from the sparse sensor arrangement envisaged by the MID approach. The data models developed by MID take various measured predictors, generally environmental variables, and determine the relationship between these variables and the natural frequency of the bridge. When this relationship is determined, we can use the predictors to predict future natural trends and then compare them to the measured natural frequencies from the SHM system in the way described in the concept above.

## 3. Data Collection

### 3.1. Bridge Descriptions

This section will briefly describe the UK bridges used in this study. To examine the robustness of the proposed MID approach when choosing the bridges for this study, a mixture of different span lengths, span numbers, and construction types was desired. The four bridges chosen have span lengths ranging from 8.9 to 98 m with span numbers ranging from 1 to 3. This represents a good sample of the common span lengths encountered on short- and medium-span bridges. The construction type/material of these bridges also varied as the sample includes a steel bridge, reinforced concrete bridges, and a steel–concrete composite bridge. One bridge, a 98 m steel tied-arch structure, was chosen so that a bridge toward the upper end of the short and medium bridge span range was included.

#### 3.1.1. Bridge 1

**Figure 2:**
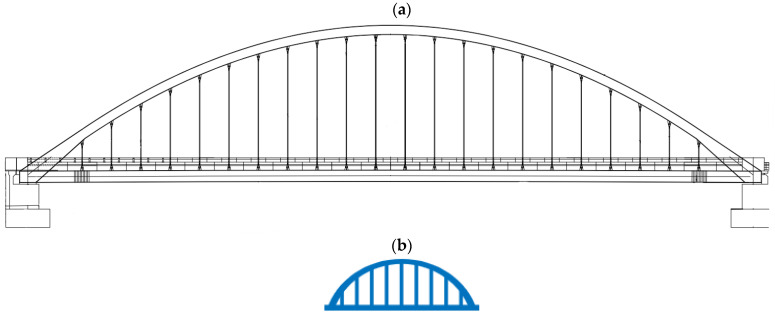
(**a**) Elevation drawing of Bridge 1. (**b**) Icon representing Bridge 1.

Bridge 1 is a steel tied-arch bridge that spans 98 m and is 27.7 m wide. Each of the main arches supports 22 tie-rod hangers with a diameter of 90 mm. These hangers support the main deck structure. The main longitudinal beams are a mix of welded plate girders (in the centre) and box sections (at the springings) with a depth of approximately 1650 mm. The 275 mm deep concrete deck acts compositely with the transverse crossbeams, which are supported by the main longitudinal beams. The steel bridge structure is supported by two concrete abutments. Figure 2a shows a drawing of the bridge’s elevation, and Figure 2b shows an icon that will be used to represent Bridge 1 in this paper. The bridge icons (one for each of the bridges), along with their colours, will be used as a convention for this paper so that when data are presented in later sections, it is easy for the reader to identify which bridge the data are from. Similarly, the colour used for plots of data from a given bridge will be consistent with the colour of the icon, making it easier to compare each bridge.

#### 3.1.2. Bridge 2

**Figure 3:**
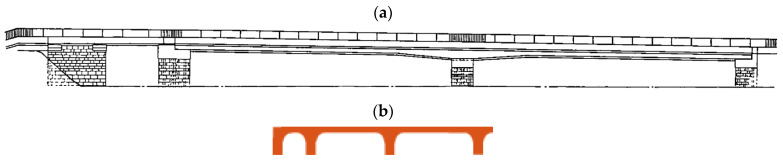
(**a**) Elevation drawing of Bridge 2. (**b**) Icon representing Bridge 2.

Bridge 2 is a three-span composite concrete and steel bridge. The bridge consists of two larger spans of 33.5 m and one smaller end span of 8.9 m. The smaller span is constructed from pre-stressed inverted T beams, which are topped by a reinforced concrete deck. The two larger spans are constructed by encasing seven preloaded steel beams in concrete (preflex method) to form the deck structure. The preflexed beams have a depth of 920 mm. Due to the length of the preflexed beams, they were spliced together during construction with high-strength, friction grip bolts. The deck elements are supported on two reinforced concrete piers supported by bored piles. Figure 3a shows a drawing of the bridge elevation, and Figure 3b shows an icon that will be used to represent Bridge 2 in this paper.

#### 3.1.3. Bridge 3

**Figure 4:**
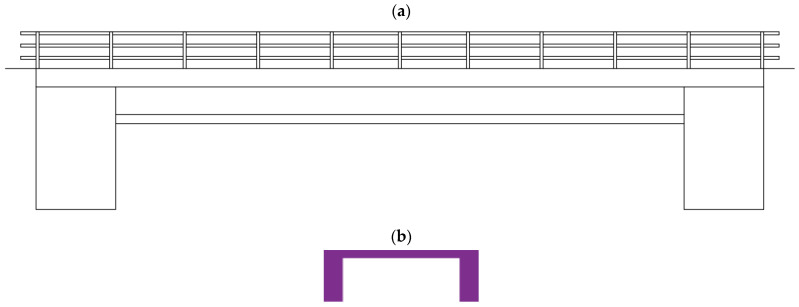
(**a**) Elevation drawing of Bridge 3. (**b**) Icon representing Bridge 3.

Bridge 3 consists of a single 32 m span. The bridge supports a dual carriageway (two lanes, unidirectional) and a footpath. The bridge is constructed from 13 concrete Y beams supporting a reinforced concrete deck. Adjoining the bridge, and supporting an adjacent dual carriageway, is an older masonry arch bridge. These two bridges are constructed with no apparent gap between them, so the extent to which one side of the deck of the concrete bridge is restrained is unknown. Figure 4a shows a drawing of the bridge elevation, and Figure 4b shows an icon that will be used to represent Bridge 3 in this paper.

#### 3.1.4. Bridge 4

**Figure 5:**
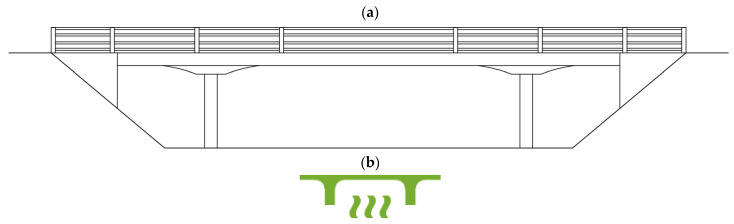
(**a**) Elevation drawing of Bridge 4. (**b**) Icon representing Bridge 4.

Bridge 4 consists of two side spans (11 m) and a central suspended span (17 m). The side spans are constructed from reinforced concrete slabs. These side spans support the drop-in centre span via a cantilever slab section. The drop-in span is constructed from precast, pre-stressed inverted T beams with in situ concrete placed over and between the beams to form a solid composite deck slab. Figure 5a shows a drawing of the bridge elevation, and Figure 5b shows an icon that will be used to represent Bridge 4 in this paper.

### 3.2. Modal Analysis

Modal tests were undertaken on the four bridges to determine the dynamic behaviour of the bridges and to ensure the natural frequencies that are measured with the SHM system are accurate and relate to the mode shapes of the bridges.

**Figure 6:**
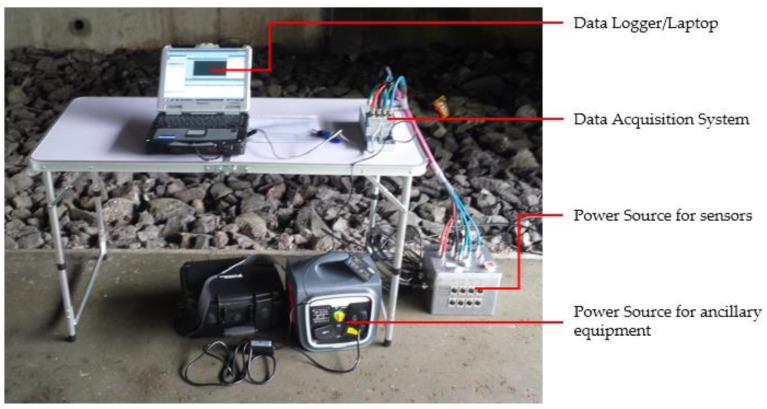
Data logger and all ancillary equipment used during the modal test.

Modal tests on the four bridges were generally undertaken using the same methodology. The modal test generally consisted of placing wired accelerometers on the surface of the bridge and recording the deck acceleration during normal bridge operation. The accelerometers used to collect the data during the modal test were high-performance, solid-state sensors designed to measure low-level acceleration (noise level 0.00025 m/s^2^/√ Hz). The accelerometers’ signals were carried to the data logger, which was located away from the general public. Figure 6 shows the datalogger and all ancillary equipment used during the modal test.

As only seven wired accelerometers were available during the modal tests and up to seventeen locations required monitoring (depending on the bridge), the data were collected in a number of stages/‘swipes’. One swipe consisted of placing all seven accelerometers and collecting data for approximately 45 min. After a swipe is complete, six of the accelerometers are moved, leaving one accelerometer in place as a reference, and another 45 min of data are collected. This swipe process was repeated until all the required locations had been monitored.

The same philosophy was adopted on Bridge 1 but due to logistical issues with access, the approach was amended to include some standalone accelerometers, which were synchronised manually. This process was described in greater detail in [16].

For the four bridges, the modal identification technique used was stochastic subspace identification (SSI). The data from the different swipes were combined by normalising the data for each swipe using the data from the reference accelerometer, which remained in the same location for the duration of each test.

The mode shapes and their associated natural frequencies can be seen in Table 1. Each column in the table represents one of the four studied bridges, and each row shows sequential modes. The natural frequency and damping ratio of each of the models are also stated.

### 3.3. Description of the Long-Term SHM System

As a single accelerometer was used to collect the long-term vibration data, its position was an important consideration, as shown in [17]. Other studies, such as [18], use numerical simulations to optimise the position of the sensors. In this work, the position of this sensor was designed to be installed at a location with some modal amplitude for all the modes. As MID tracks the natural frequencies of the structure to identify changes in behaviour, any modes that cannot be tracked due to the placement of the sensor may mean that certain damage cases may not be detected. Ideally, the sensor should be placed close to the point of maximum modal amplitude. However, for a given bridge, it is unusual for this point to be the same for all modes. So, for example, for Bridge 2, the midspan of span 1 would be a very suitable location to place the sensor to capture information on modes 1–4, as the midspan happens to be the anti-node for these modes. However, the midspan would be a very poor location to capture information on mode 5, as the midspan happens to be the node point for mode 5. In most cases, this results in a trade-off, and it was found that positioning the sensor close to the quarter-span point on all four bridges allowed the sensor to detect the most modes/frequencies.

The SHM system was designed to be as general and as easy to install as possible. As such, the accelerometer can be installed on the surface of the bridge deck, with the variations in the bridge types being accounted for in optimising the placement of the accelerometer, which was discussed above. The MID method then takes further steps to account for variation in the bridges, such as selecting the best predictors (discussed in Section 5) for the data models and testing different outlier detection methods.

**Figure 7:**
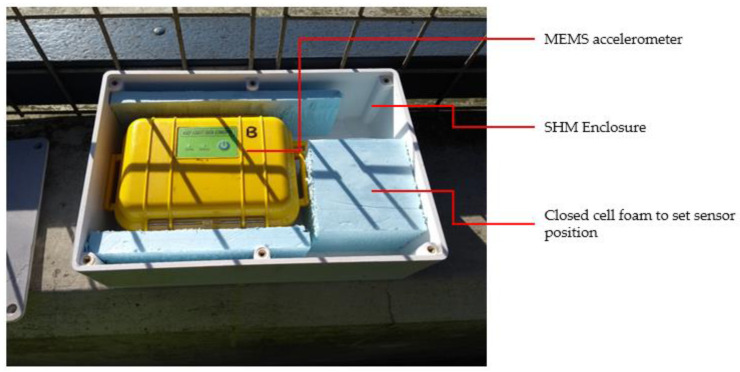
Monitoring enclosure with the MEL accelerometer.

The SHM system used to obtain the long-term monitoring data consisted of one MEMS accelerometer and one environmental sensor. The accelerometer used was the ‘Multifunction Extended Life (MEL) Data Logger’ from Gulf Coast Data Concepts. This accelerometer measures acceleration in three axes within a range of ±2 g and has a real-time clock to timestamp every acceleration measurement. The acceleration data from the sensor are stored locally on an SD card at a sampling rate of 128 Hz, and the sensor is powered by an internal battery. The MEL accelerometer was housed in an enclosure that was in turn attached to the deck of each of the bridges. One of the enclosures can be seen in Figure 7.

The environmental variables were measured with an environmental sensor. This single sensor was capable of measuring both air temperature and humidity. The sensors were not placed within the enclosure to avoid inaccurate temperature measurements due to solar gain. Instead, the temperature and humidity sensors were placed out of direct sunlight, giving a representative value of local air temperature. The location of the temperature sensors varied on each of the four bridges. However, they were typically placed on the abutment shelf or at the base of an abutment out of direct sunlight.

## 4. Review of Collected Data

This section presents an overview of the acceleration and temperature data collected from all four bridges before undertaking the data-modelling process. This review ensures that the data are credible so that they can be used in the data modelling process.

### 4.1. Acceleration Data

**Figure 8:**
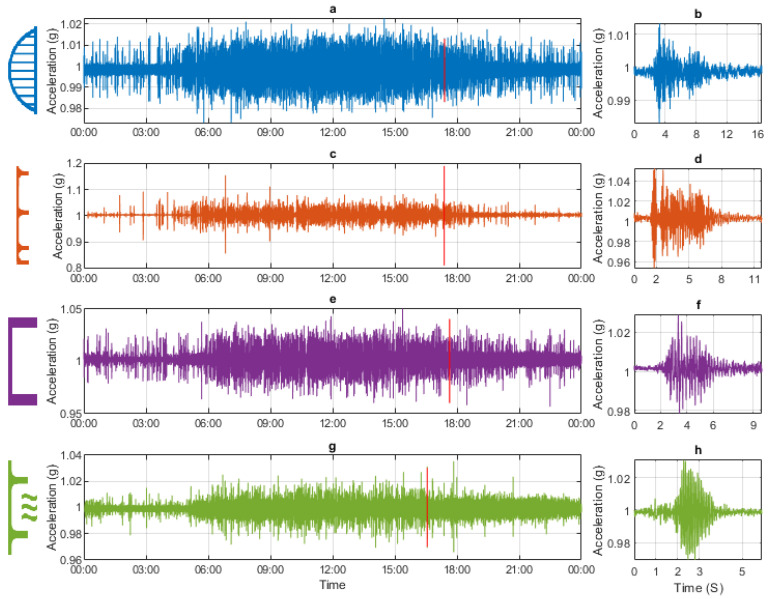
Typical example of acceleration data. (**a**) Bridge 1: 24 h. (**b**) Bridge 1: single loading event. (**c**) Bridge 2: 24 h. (**d**) Bridge 2: single loading event. (**e**) Bridge 3: 24 h. (**f**) Bridge 3: single loading event. (**g**) Bridge 4: 24 h. (**h**) Bridge 4: single loading event.

Typical acceleration data from the four monitored bridges are shown in Figure 8. Figure 8a shows acceleration data over 24 h for Bridge 1. Figure 8b shows the acceleration signal for a single-vehicle event that occurred during the 24 h (indicated by a red line in Figure 8a). Figure 8c,e,g show the acceleration signal collected from Bridges 2, 3, and 4, respectively, over the same 24 h period (as shown in Figure 8a). Figure 8d,f,h show a single-vehicle event within the 24 h from Bridges 2, 3, and 4, respectively (location marked with a red line in the corresponding 24 h plot).

In the 24 h plots (Figure 8a,c,e,g), all four bridges show the same pattern. Most activity occurs between 06:00 and 21:00, with less activity during the night. This pattern is expected with the higher traffic generally occurring in the daytime. By looking at the y-axis limits, it is evident that the range of acceleration for Bridges 1, 3, and 4 is approximately similar, with accelerations typically in the range of 1.05 g to 0.95 g. This is due to the standard deviation of the 24 h acceleration signals being similar for Bridges 1, 3, and 4 (0.0014 g, 0.0014 g, and 0.0011 g). The range of acceleration experienced by Bridge 2 is slightly higher, with accelerations typically in the range of 0.9–1.1 g and a standard deviation of the 24 h acceleration signal of 0.0027 g. The higher standard deviation would suggest that Bridge 2 experiences a slightly higher magnitude of acceleration.

The single-vehicle events (Figure 8b,d,f,h) all show a characteristic free decay and look credible, with the duration of the free decay periods being approximately similar. The free decay periods being of a comparable duration is consistent with the similar levels of damping observed in the modal analysis (Table 1).

### 4.2. Temperature Data

**Figure 9:**
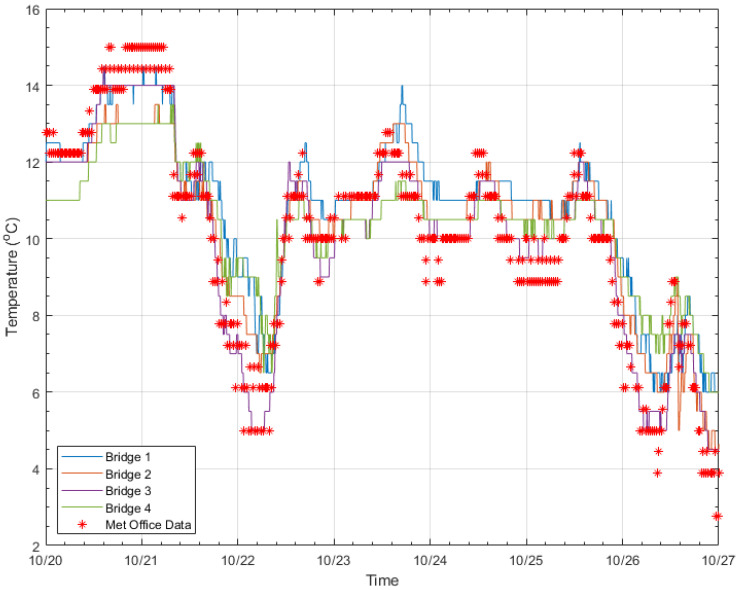
Temperature data over 7 days from each of the monitored bridges and met office data for the same period.

To ensure that the collected temperature data were sensible/credible, they were compared to data from a nearby weather station. This validation of the air temperature data is an important step, as the temperature data will be used as the main predictor in each of the data models used to predict the natural frequencies of the bridges. Figure 9 shows seven days of temperature data, with the blue, orange, purple, and green lines representing Bridges 1, 2, 3, and 4, respectively. Temperature data between each of the bridges are fairly consistent, albeit with slight differences, depending on the location of the bridge. The corresponding air temperature from the weather station is also plotted (red star markers). This met office weather station ranges from 11 to 14.5 miles from the four monitored bridges. The broad temperature trends at all four of the bridges match well with the met office air temperature. There are some small differences, e.g., for the hottest and the coldest temperatures, the met office temperature tends to be fractionally higher/lower, but this is likely due to the met office temperature being taken in a weather station, whereas the temperature at the bridges is taken close to a structure, which could account for the slight differences. Overall, the data presented in Figure 9 suggest that the air temperature readings collected from the long-term monitoring system are credible.

### 4.3. Frequency Data

#### 4.3.1. Time Series Frequency Data

The first step in the frequency extraction process was to split the collected acceleration data into 30 min sections. A stochastic subspace identification (SSI) method was used to convert time-series data to frequency data. A unique set of SSI inputs for each identified mode was determined using the method outlined in [19].

**Figure 10:**
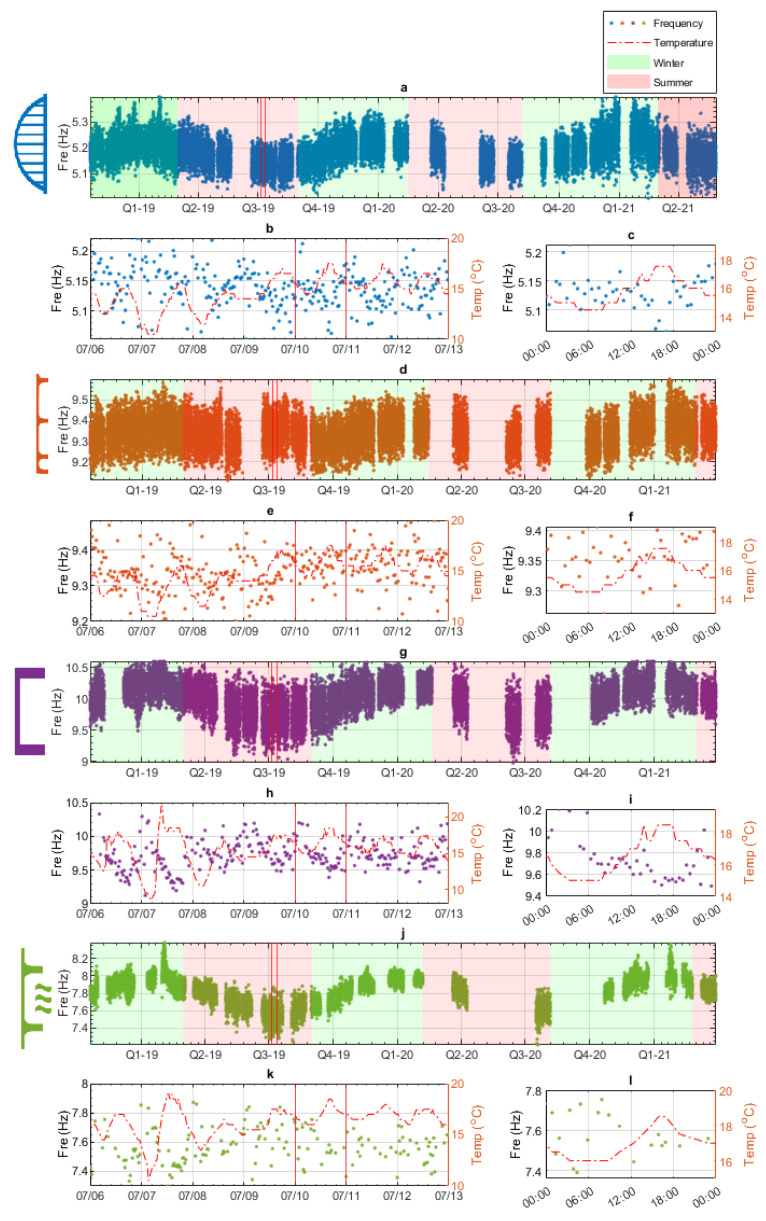
Example of natural frequency data from four bridges. (**a**,**d**,**g**,**j**) Natural frequency over 30 months; the summer and winter are indicated. (**b**,**e**,**h**,**k**) Natural frequency over 7 days and corresponding temperature. (**c**,**f**,**i**,**l**) Natural frequency over 1 day and corresponding temperature.

The data were recorded between October 2018 and May 2021. However, there were some breaks in the data due to the availability of personnel for data collection and the COVID-19 pandemic. An example of the natural frequency datasets for Bridges 1–4 is presented in Figure 10. Figure 10 presents the four highest natural frequencies, one for each of the monitored bridges. The highest natural frequency was chosen, as they typically show the highest variation with time/temperature. Figure 10a shows the mode 5 frequency data over the entire 30-month monitoring period for Bridge 1. The frequency data are shown with the blue dots and, for convenience, the colder/winter months (September–February) are shaded in green, and the warmer/summer months (March–August) are shaded in red. There are occasional gaps in the data due to delays in collecting the data or changing the batteries. The most significant gaps occur in Q2 and Q3 2020, resulting from restrictions on travel/site work due to COVID-19. Figure 10b shows the same mode 5 frequency data over seven days and the corresponding temperature data, with frequency and temperature being plotted against the left and right y-axes, respectively. The seven-day period is indicated by the two red lines in Figure 10a. Figure 10c shows one day of frequency and temperature data, and this interval is shown in Figure 10b with the two red lines. The resolution of the frequency data are generally two readings per hour, and Figure 10c shows how this fine resolution of the data can be used to observe changes over short periods of time, e.g., a few hours. The same format is used to present the data for the remaining three bridges. The data from the highest mode for Bridges 2, 3, and 4 are presented in Figure 10d–f, g–i, and j–l, respectively.

#### 4.3.2. Relative Variation

While the behaviour of a given modal frequency with respect to temperature can be appreciated by looking at the time series plots such as Figure 10, further useful insight can be obtained by looking at some statistical metrics. For example, the relative variation of each of the natural frequencies gives an appreciation of how much normal variation occurs due to operational and environmental effects. This metric has been used in previous studies, such as [20]. The relative variation is defined using Equation (1).

(1) $${RV}_{i}=\frac{f_{max}-f_{min}}{f_{mean}}\times 100\%$$

where $f_{max}$, $f_{min}$, and $f_{mean}$ are the maximum, minimum, and mean of the extracted natural frequency dataset. Table 2 shows the relative variation for all the identified modes. The relative variation ranges from 4.87% of the second mode of Bridge 2 to 16.41% of the first mode of bridge 1. To allow the levels of variation to be more easily visualised, the variation percentages are colour coded in the table (blue 17–13%, green 13–9%, red 9–4%). This level of variation is consistent with the levels reported in previous studies. Ref. [6] studied 12 modes associated with a footbridge and found that the relative variation ranged from 14 to 20.6%. Ref. [21] found the relative variation of a stress ribbon footbridge’s frequencies was between 15.3% and 21.4%. Ref. [3] observed that the normal environmental change accounts for the variation in extracted frequencies of 0.962–6.690% for the first eight modes for a multi-span cable-stayed footbridge bridge. While the studies quoted above do not study highway bridges, the range of relative variation from other short-span bridges is consistent with the findings here.

#### 4.3.3. Relationships between Variables

**Figure 11:**
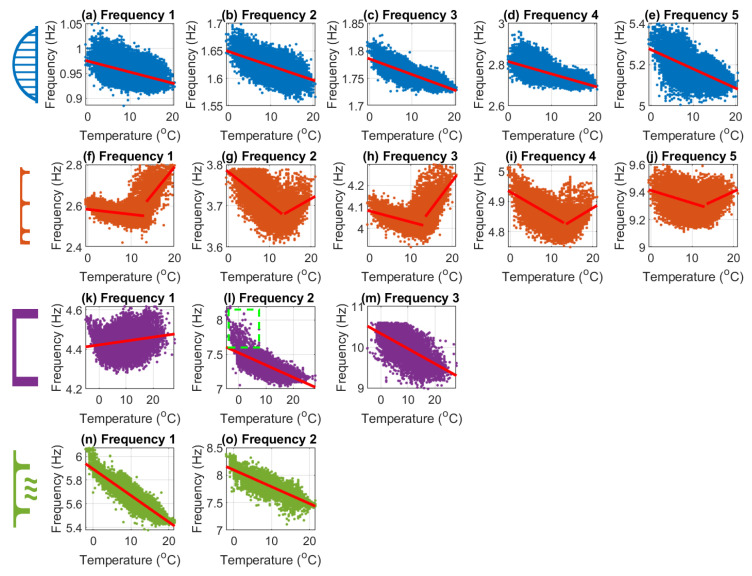
Frequency vs. temperature data (all bridges and all frequencies).

Before creating the data models, it is important to identify the approximate relationships between the natural frequency data and environmental data. Identification of the specific relationships will inform what type of regression will most accurately model the collected data. For example, if the relationships were non-linear, using a linear regression would not accurately model the data. Figure 11 shows the air temperature (x-axis) and corresponding natural frequency (y-axis) plotted for each of the identified modes.

For Bridges 1, 3, and 4, the majority of the plots in Figure 11 show frequency and temperature having an inverse relationship, as expected. To highlight this fact, the best linear fit line is shown for each plot. It can be seen that most have a downward slope, indicating an inverse correlation. The first mode for Bridge 3 shows a slight positive correlation; however, the magnitude of the positive slope is quite small. In some modes, sub-zero temperatures cause a weak bilinear relationship between frequency and temperature. The mode where this is most obvious is mode 2 for Bridge 3; this is highlighted by the green dashed box in the top left of Figure 11i. This kind of bilinear behaviour around 0 °C is similar to that observed on the Z24 bridge [10] due to freezing events and is felt to be related to the effective stiffening of the deck when water in the deck/surfacing freezes.

The plots for all modes of Bridge 2 (orange) are noticeably different from the other bridges, as they display a distinct bilinear relationship between frequency and temperature, with the pivot point being approximately 13 °C. Due to the limited number of studies that have carried out long-term monitoring on short- to medium-span bridges, there are very few examples of bilinear behaviour, such as that being exhibited by Bridge 2 at 13 °C. The closest example is the bilinear behaviour of the Z24 bridge [10] due to the freezing events mentioned earlier. However, no one has reported bilinear behaviour of short/medium-span highway bridges not related to freezing. Considering the very small sample size of short- and medium-span bridges internationally that have undergone long-term frequency monitoring, this is perhaps not surprising. However, as this behaviour is not expected, steps were taken to ensure that this was not a measurement error. The first step was to ensure that the anomalous behaviour was measured throughout the entire monitoring period. This ensures no time-dependent fault in the sensor, such as incorrect repositioning after a battery replacement. This check showed that measurements were distributed across the entire monitoring period. The second step was to check the long-term sensor against other reference sensors. This was performed over 1 h with two additional MEL accelerometers (other than the long-term sensor) and two high-precision wireless sensors. All sensor’s acceleration and natural frequency measurements agreed with the long-term sensor, indicating that the sensor was giving accurate measurements. After the frequency was extracted from all five sensors using the same method, the extracted frequencies showed no variations. Having established that the pattern being observed was genuine (i.e., not due to experimental error), time was spent trying to identify the source of this behaviour. Ultimately, the specific source of the behaviour was not established. However, of the possible sources of the behaviour, a change in boundary conditions occurring at 13 °C was considered the most plausible/likely cause, or at least one that should be investigated further. Unfortunately, checking this would require further field work, e.g., instrumenting the bridge to track bearing movements, which was not possible in the timeframe of this study.

The observed frequency–temperature relationships discussed above have implications for the data modelling process. The sub-zero behaviour of the bridges (mainly Bridges 2 and 3) can, for the most part, be ignored, as the portion of the dataset corresponding to sub-zero temperatures is very small (approximately 1.3%). If a significant amount of data were related to sub-zero temperatures, two data models would be required, one for above zero degrees and one for below zero degrees. However, in this dataset, only a minimal amount of sub-zero temperature events occurred, and so only one data model is required. One necessary caveat is that the data model will be unreliable at predicting the behaviour during sub-zero temperature events.

The bilinear relationship, which was observed in the Bridge 2 data, may have a more significant effect on the accuracy of the data models. There are two options that were considered; the first was to separate the data between temperatures below 13 °C and temperatures above 13 °C and create two separate data models. The second was to create a data model with all of the data and combine this with a GPR regression, possibly resulting in a slight reduction in the model’s predictive power. Both of these options were tested when developing the data models. However, it was determined that the amount of data above 13 °C (approximately 20%) was insufficient to provide accurate results. Therefore, the data models created for each mode of Bridge 2 were developed using all of the data, and the prediction capability was found to be acceptable. This was due to the GPR method offering a universal approximator when estimating relationships between variables.

## 5. MID Method Overview

**Figure 12:**
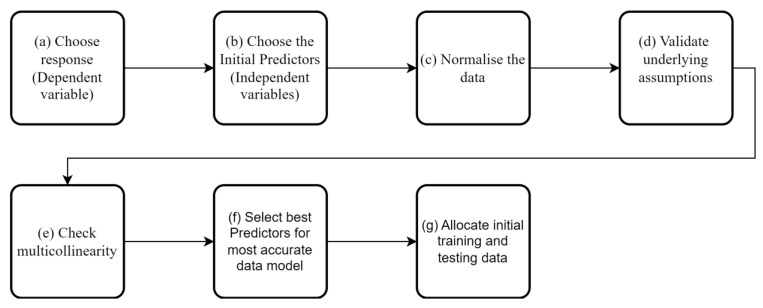
Flowchart showing the workflow for MID.

The MID process is described in [15], but in this section, we will provide an overview. Many SHM studies have used data modelling [22,23,24,25], but no workflow for modelling sparse data collected from short- and medium-span bridges has been presented in the current SHM literature. Although general principles of data modelling are known, each dataset has subtle differences, and thus, a workflow that works for the sparse sensing data envisioned by MID needs to be developed. The data models used in other studies have been trained with large datasets (e.g., those available on large cable-supported bridges), which differ in approach from the ones used here. The workflow developed in [15] is presented in Figure 12. It includes considerations that should be made when using a limited dataset in conjunction with a data model and, in particular, a regression analysis.

As shown above, when creating a data model using regression techniques, several factors need to be considered. The below points give a brief overview of the stages that are considered before training the data models.

**(a)**  **Choose dependent variable:** This is the variable of interest, in this case, the natural frequency of the bridge, e.g., the natural frequency of a given mode.
**(b)**  **Choose independent variables:** These variables are used to predict the dependent variable. When using MID, a balance needs to be struck between having predictors (e.g., temperature) that sufficiently influence the dependent variable while also being easy to measure with low-cost equipment. The predictors that were chosen for this study are as follows:
  Air temperature at the time of frequency measurement;
  Air temperature both 1 and 6 h prior to frequency measurement to capture any potential thermal lag of the material/bridge structure;
  Humidity, which has been shown in some studies [26] to impact the natural frequency of structures.
**(c)**  **Normalisation of the data:** While not all regression methods require the data to be normalised, linear regression methods are affected by non-normalised data. The normalisation of the data can also be necessary when determining which of the predictors is the most influential in the data models. For this process, the dependent variable and all independent variables have been normalised.
**(d)**  **Validate underlying assumptions:** Before any regression analysis, the following assumptions for the data need to be validated:

- Linearity of the phenomenon measured (for linear regression);
- Constant variance of the error terms;
- Independence of the error terms;
- Normality of the error term distribution.

Testing of assumptions I to III is usually performed by plotting residuals against predicted responses. The distribution of the resulting graph allows for the checking of any assumption violations. Details of how to check the assumptions can be found in [27]. The last assumption (IV) is the normality of the error term distribution, which is checked by plotting the residual distribution.

**(e)**  **Multicollinearity:** Multicollinearity occurs when independent variables in a regression model are correlated with each other. If this happens, it is hard to isolate the relationship between each of the independent variables and the dependent variables. Multicollinearity will generally weaken the predictive power of the regression model. Multicollinearity is typically more of an issue when many variables are being studied. In this paper, there are relatively few variables. However, several of the variables are temperature-based, and the likelihood that these variables will be correlated is high, so it is essential to ensure they will not adversely affect the data model.
**(f)**  **Select best predictors:** Removing independent variables with little predicting power is important for several reasons as follows:

- It reduces the time needed to train the regression.
- It reduces the complexity of the regression analysis.
- It allows the analyst to gain a better understanding of the behaviour of the structure. Filtering out the less important factors identifies which of the remaining factors are most strongly correlated to the dependent variable’s observed behaviour.

There are many different methods available to aid feature selection in a regression context. Here, a relief-based approach is adopted; relief-based approaches avoid the need to carry out an exhaustive comparison between candidate features, instead relying on a nearest neighbour type measure to assess inter-relationships. ReliefF [28] is a variant that assigns weights to the predictors given a set of observations.

**(g)**  **Allocate initial training and testing data:** The next step is to decide how much data to use for the training of the model and how much to use for the testing of the model. To have the best predictive power, the model will most likely need to be trained with ‘one cycle of data’. These data should include all expected ranges of temperatures.

Once the above steps are complete, the first iteration of the data model is complete. It is also necessary to check how outliers in the dataset affect the model.

## 6. Refinements to MID: Automation and Consistency

To be able to operate across a range of bridges, some refinement of the Minimal Information Data-modelling (MID) method is required to achieve automation and consistency, as these factors are crucial for its scalable utility across an entire bridge network. Ensuring consistency in the application of MID is essential to facilitate the possibility of widespread implementation and to streamline the data modelling process. Automation simplifies and standardises the implementation process, making it more efficient and reducing the likelihood of errors.

One effective approach to achieve automation and consistency is through the development and utilisation of software, programs, or scripts tailored for MID implementation. To this end, MATLAB (R2019a) was used to produce tools that can offer several functionalities to streamline the process.

- Automated Outlier Detection: The scripts can automate outlier detection using various methods, facilitating comparisons of data before and after outlier removal. This enables informed decisions regarding the inclusion or exclusion of outliers in the analysis.
- Regression Assumption Checking: The scripts can assess regression assumptions of the predictors and provide functions to transform the data if they do not meet these assumptions. This ensures the validity of the regression analysis and enhances the reliability of the results.
- Multicollinearity Check: The scripts can check for multicollinearity among predictor variables and offer functions to remove data that does not pass the check. Addressing multicollinearity enhances the robustness of the data models generated by MID.
- Data Splitting: The scripts can automate the process of splitting the data into training and testing pools.
- Regression Methods Selection: The scripts can provide functions to undertake regression using different methods, allowing users to input their preferences. This flexibility enables users to explore various regression techniques and select the most suitable approach for their specific bridge assessment needs.
- Comparison Metrics Display and Saving: The scripts can display and save comparison metrics from the resulting data models, enabling users to compare the performance of different regression methods. This functionality facilitates informed decision making and enhances the transparency of the assessment process.

## 7. Application of MID to the Studied Bridges

In this section, the data collected from the four bridges will be used to validate the data modelling process described in Section 5 with the automation refinements described in Section 6.

The primary purpose of the data model is to remove the environmental and operational effects from natural frequency data so that a potential change in the condition of the bridge can be identified. As long as the future environmental variable stays within the range of environmental variables that the data models are trained, then it is expected that reliable predictors will be made. If, in the future, there are extreme environmental conditions, such as extreme temperatures, then the data models may need to be retained with the new environmental conditions to give reliable predictions. The data models created in [15], trained on data from a steel road bridge, have shown that they can remove enough of the environmental effects to identify possible damage using the residuals. Here, the data modelling process developed in [15], including the refinements described in Section 6, will be trialled on data from differing bridge types (varying construction type and span). This will show if the MID process is robust enough to remove sufficient environmental effects so that the magnitudes of frequency shift known to indicate potential damage can be detected.

Section 7.1 presents the results of two data models out of a total of fifteen (one for each natural frequency identified). Due to some unusual behaviour found in one mode of one bridge, Section 7.2 investigates the collected data to determine the cause of this discrepancy. Finally, Section 7.3 and Section 7.4 present and utilise a standardised method to determine the level of frequency shift that all the data models can detect.

### 7.1. MID Results

In this section, two figures are shown to give an example of the data model results after the MID process outlined in Section 5. After each of the data models (one for every natural frequency) has been trained, every observation has the extracted frequencies resulting from the SHM system and the predicted natural frequencies resulting from the data model. The residuals are determined for each observation and are defined as the difference between predicted values for the natural frequency and the natural frequency obtained from the SHM system.

**Figure 13:**
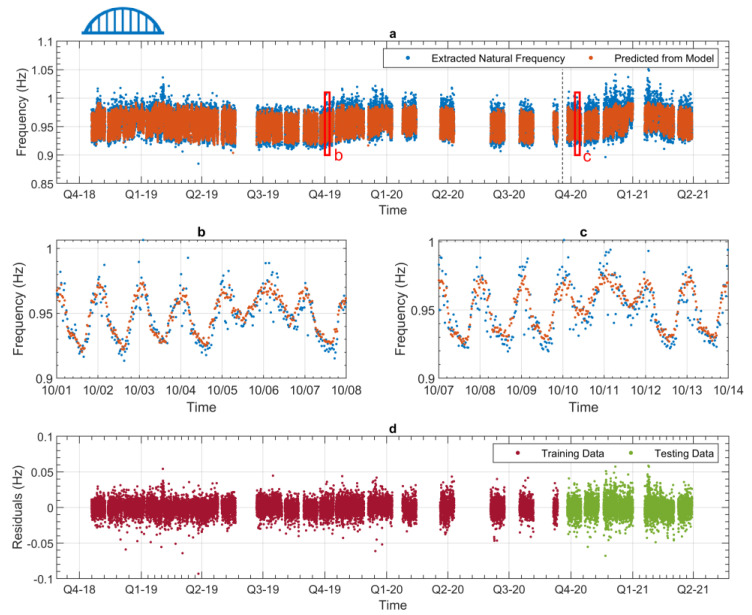
Results from a data model (Bridge 1—1st frequency). (**a**) Full experimental dataset and corresponding values predicted by the regression model (divided into training and testing). (**b**) Zoomed-in view showing approximately 1 week of data during the training phase. (**c**) Zoomed-in view showing approximately 1 week of data during the testing phase. (**d**) Training and testing residuals across the whole monitoring period.

**Figure 14:**
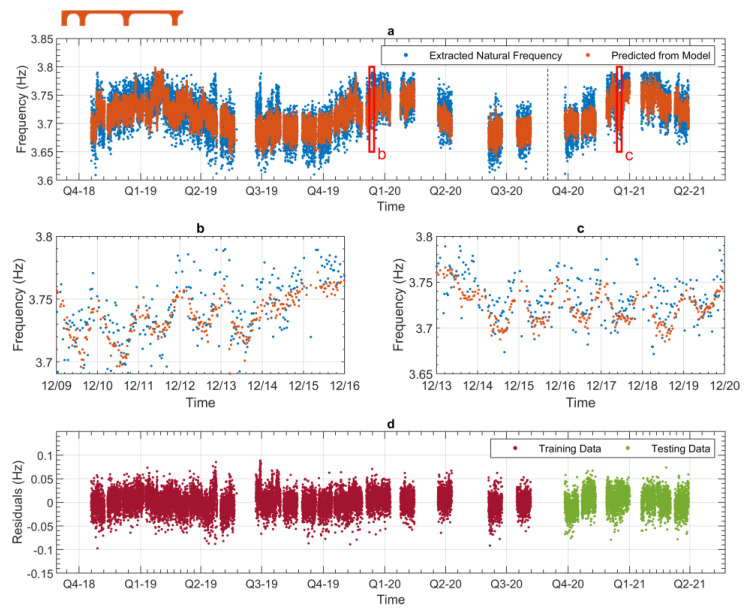
Results from a data model (Bridge 2—2nd frequency). (**a**) Full experimental dataset and corresponding values predicted by the regression model (divided into training and testing). (**b**) Zoomed-in view showing approximately 1 week of data during the training phase. (**c**) Zoomed-in view showing approximately 1 week of data during the testing phase. (**d**) Training and testing residuals across the whole monitoring period.

Figure 13 presents the frequency with the highest relative variation (seen in Table 2), for Bridge 1, mode 1. Figure 14 presents the lowest relative variation frequency (Table 2) for Bridge 2, mode 2. Both Figure 13 and Figure 14 are in the same format. In plot (a), the extracted frequencies are shown with the blue dots, and the predicted frequencies from the data model are shown with the orange dots. The black dashed vertical line separates the training and testing data. Plots b and c zoom into one week of training and testing data, respectively. The periods that are shown in plots b and c are indicated with the red boxes in plot a. Plot d shows the residuals of the data model against time.

Figure 13 and Figure 14 can give us an indication of the performance of the data models. When studying these plots, we can look at how accurately the data models can predict the variations in the original data. In this case, our variations are the annual and daily changes to the natural frequency. Figure 13 and Figure 14 show that the performance of both data models presented is similar, i.e., that the variations in the data can be predicted in both cases. Figure 13a and Figure 14a show that the data models can accurately predict the annual trends of both frequencies. Figure 13b,c and Figure 14b,c show that the daily frequency trends are also accurately predicted by the models. Figure 13c and Figure 14c show that the testing data are of particular interest, as these plots show how well the data models can predict future behaviour. This is the case because the testing data have not been used in the training process, and so if the data model can predict the testing data, it should be able to predict future behaviour, which it also has not been trained on. It should be noted here that the performance of the testing data from both models is comparable to the training data. This indicates that the predictive power of the data models has been maximised [27]. Figure 13d and Figure 14d show the difference between the predicted values and the extracted values, i.e., the residuals. Viewing the residuals in this manner allows the comparison of model performance between the training and testing data. This figure indicates no periods of significantly better or worse prediction ability for either of the data models. As previously discussed in Section 4.3, the modes for Bridge 2 demonstrated some bimodal behaviour, depending on whether the temperature was above or below 13 °C. However, it can be seen in Figure 14 that the data model was able to account for this, as the residuals shown in Figure 14d are relatively consistent across the full monitoring period. Figure 13 and Figure 14 are representative of what the data models for the other modes look like, so to avoid repetition, only these two are shown here.

The overall goal was to use the data model to identify a change in stiffness. For this to be possible, the data models must also be able to identify stable behaviour. Based on basic visual inspections during the site visits, it is assumed that the bridges have not developed damage during the monitoring period. Therefore, the 15 data models should reflect this behaviour. Table 3 shows the distribution of the training and testing residuals (in Hz) for each of the identified modes. The training residuals are shown with blue bars, and the testing residuals are shown with orange bars. For example, the distributions plotted for mode 1 of Bridge 1 (top left of the table) are simply plotting the training and testing residuals previously shown in Figure 13d. Similarly, the training and testing residuals shown for mode 2 of Bridge 2 in Table 3 are simply a histogram plot of the training and testing residuals in Figure 14d. Table 3 shows that the testing data from all but one of the data models (orange bars) fall within the distribution of the training data. This indicates that the behaviour of the bridges remained unchanged over the monitoring period. Mode 4 of Bridge 1 shows some deviation; the distribution of the testing data does not have the same distribution as the training residuals. The discrepancy in the distributions shown is only slight, but it can be seen that the testing residuals are shifted to the right of the training residuals. This behaviour will be investigated in Section 7.2 to try and identify the cause of the discrepancy between the training and testing residuals. Table 3 shows that each of the other 14 data models has identified stable behaviour. The sensitivity to damage of the data models is still unknown. An indication of the sensitivity can be obtained by looking at the range of the residuals (shown on the x-axis in the figures). The smaller the range of the residuals, the increased likelihood that the data model will be able to detect small changes in frequency. For example, mode 1, Bridge 1 has a range of ±0.03 Hz, whereas the range of the residuals for mode 1, Bridge 3 is ±0.1 Hz, so it is likely that a smaller frequency shift will be detectable in mode 1, Bridge 1 than in mode 1 of Bridge 3. Looking at the range this way is a crude indicator, so Section 7.4 will identify, in greater detail, the level of frequency shift that can be determined.

### 7.2. Study of Bridge 1, Mode 4 Discrepancy in the Residuals

**Figure 15:**
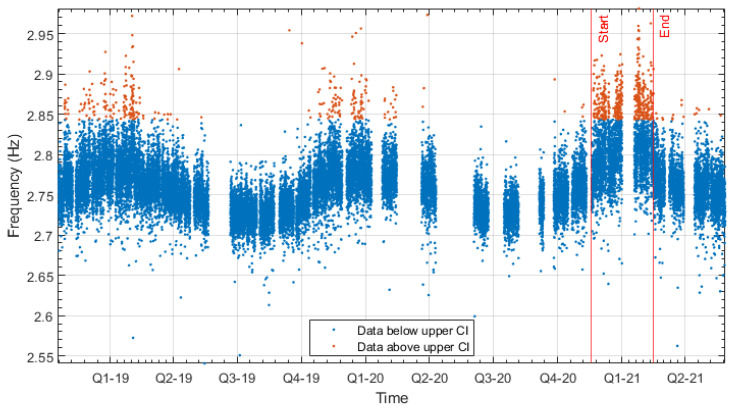
Frequency 4 from Bridge 1 over the entire monitoring period; the highest frequencies are highlighted.

In this section, the discrepancy that was observed in Table 3 between the distributions of the training and testing residuals of mode 4 of Bridge 1 will be investigated. This can be seen both in the residuals of the data model and in the natural frequency data presented in Figure 15. This figure shows the fourth mode frequency for the entire monitoring period. To understand when the highest frequencies were observed, the data have been divided into two groups. The data higher than the upper 95% confidence interval are shown with the orange dots, and any frequency that is below the 95% confidence interval is shown with blue dots. The two vertical red lines in Figure 15 indicate the proportion of the testing data that caused the discrepancy in residuals/data, leading to the overall testing data being dissimilar to the training data. Using this plot, it can be observed that 60% of the highest frequency data (orange dots) are contained in this period, despite only accounting for 10% of the data. The fact that there is an increase in frequency in a discreet period followed by a return to normal behaviour indicates that it was likely due to a transient change in the environment as opposed to a change in the structure.

**Figure 16:**
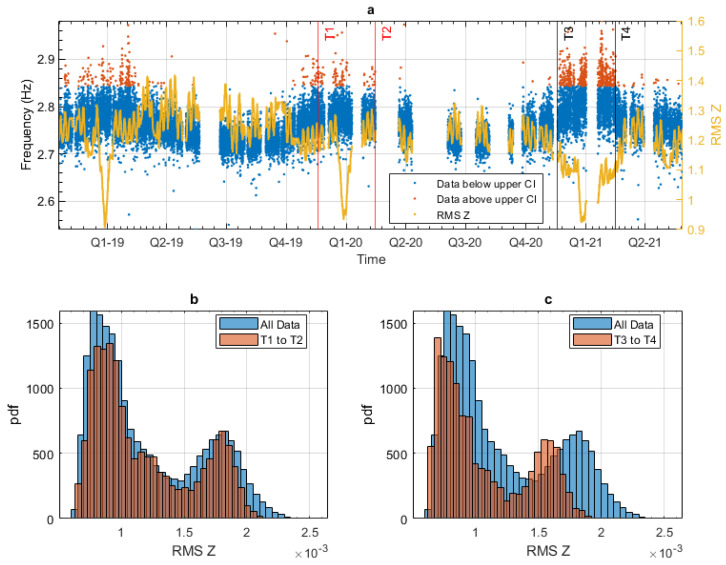
Reduction in RMS values observed during the testing period. (**a**) Frequency 4 from Bridge 1 over the entire monitoring period; the highest frequencies are highlighted. (**b**) Vertical RMS histogram for the entire period and T1 to T2 period. (**c**) Vertical RMS histogram for the entire period and T3 to T4 period.

Knowing that the majority of the highest observed frequencies occurred during the period indicated in Figure 15, the next step was to determine if any other abnormalities were observed during this period. Figure 16 presents the results of that investigation. Figure 16a shows the same data that was presented in Figure 15, including an average of the vertical RMS acceleration shown with a yellow line. The vertical RMS acceleration measures the energy in the acceleration data and will be affected by the amount of traffic on the bridge. Three downward spikes are observed in the data, all occurring at the same period each year, namely, at the end of Q4 2018, 2019, and 2020. These sharp reductions in the vertical RMS values may result from the reduced traffic during the Christmas holiday period. This behaviour is as expected; however, a more prolonged drop in the RMS values can be seen in the period of interest in the testing data (between T3 and T4 lines). This drop does not occur due to a periodic event, like the observed downward spikes due to the reduced Christmas holiday traffic. To ensure that the drop in RMS values occurring between the T3 and T4 lines is abnormal, the same period during the previous year was used as a comparison. This comparison period is indicated between the T1 and T2 red lines in Figure 16a. Figure 16b,c show the histograms of the RMS values during these two periods. Figure 16b shows both the RMS values for all the data shown with the blue bar and the RMS values during the T1 to T2 period. Figure 16c again shows the RMS values for all the data shown with the blue bar and the RMS values during the T3 to T4 period. If these two plots are compared, it can be seen that the RMS values during the first periods (T1 and T2) match the distribution of the overall data, whereas the distribution of the RMS values during the second period (T3 and T4) show a significant reduction. Figure 16c shows that there was a reduction in the RMS values during the period of the testing data, which caused the discrepancy between the training and testing data. One possible reason has been given for a reduction in RMS values: a reduction in traffic, like that shown during the Christmas period. However, no such obvious event occurred during the period in question. It was observed during site visits that traffic control measures were in place around that time. These traffic measures restricted the speed of the traffic in one direction. The exact dates of the traffic control measures are unknown, so this is speculative, but a reduction in traffic speed could reduce the energy in the acceleration signal. The reduced energy could, in turn, lead to reduced accuracy in extracting the modal frequency.

### 7.3. Method to Determine the Lowest Detectable Natural Frequency Shift Using MID

The development of a standardised testing method enables all data models to be compared fairly. A standardised method allows for the variables, such as the threshold values or the point at which the frequency shift is introduced, to remain constant. If this was not the case and the variables were changed for the testing of every data model, then smaller frequencies would be identified, but comparisons between detectable frequency shifts would not be justifiable.

**Figure 17:**
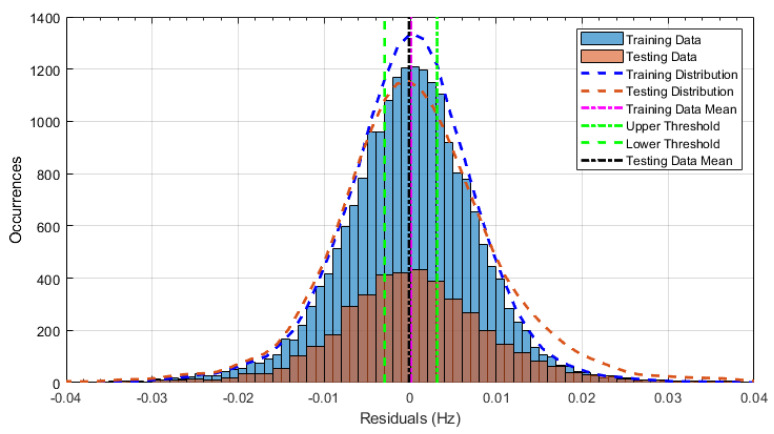
Explanation of the frequency shift detection method. Residuals from the data model training and testing with thresholds are marked.

The principle behind the developed method can be described using Figure 17. This figure shows a set of typical training and testing data residuals for the data models. The training residuals are shown with the blue bars, and the testing residuals are shown with the orange bars. As both the residual sets are normally distributed, a probability density estimate can be used to represent each residual set. The probability density estimate is a way to describe the distribution using probability and is a measure of how likely the residual is to be a certain value. Another way to visualise the probability density estimate is as a normalised histogram with the area under the curve equal to 1. The two probability density estimate curves are shown in Figure 17; the training probability density is indicated by the blue dashed line, and the orange dashed line indicates the testing probability density. Salient information, such as the mean, can be extracted from the training and testing residuals using the probability density estimate. The mean value can be obtained from the probability density estimate and used to compare the two sets of residuals. Figure 17 shows the training residual mean with the magenta vertical dashed-dot line and the testing residual mean with the black dashed-dot line. In this typical example, the two means are very similar, indicating very little difference between the training and testing residuals, i.e., stable structural behaviour. Threshold values are now chosen so that any significant deviation between the two means can be identified, which signifies a change in the testing residual from the training residuals. There is no accepted threshold value that will be optimum for every structure. If the structural behaviour of a bridge normally shows only small fluctuations, then a small deviation from this pattern may indicate an issue, whereas if a bridge normally displays large fluctuations in dynamic behaviour, a larger threshold value will be needed. For the method proposed, a degree of trial and error was used to determine suitable threshold values. The starting point was to use standard deviation as a guideline. Because the distributions were normal, the standard deviation is representative of the percentage of data between two points. After trialling a few different values, it was found that a 0.5 standard deviation on either side of the mean was a suitable threshold. This 0.5 standard deviation on either side of the mean (1 standard deviation in total) is approximately equal to 40% of the data. In Figure 17, the upper and lower threshold values are indicated with the green dashed-dot and green dashed lines, respectively. With the threshold values decided, the method will detect abnormal behaviour when the mean of the testing data exceeds one of the threshold values; in other words, when the black dashed-dot line crosses one of the green lines in Figure 17.

Using the principles described above, the following process was developed to identify the detectable frequency shift of each data model:

1. Fit a probability density estimate to the training residuals (blue dashed line Figure 17) and find the upper and lower threshold values from the distribution curve properties (two green lines in Figure 17).
2. Introduce a small frequency shift (usually 0.01 Hz) at the halfway point in the testing data. The location of the frequency shift is arbitrary so long as there is enough data before and after to ensure the data model can detect normal and abnormal behaviour.
3. Split the testing data into individual windows. The testing data are split so that testing of the residuals can be performed over short time periods, as opposed to testing all the data at once. Each window comprises 1000 points (approximately 20 days) and has a 70% overlap. A total of 1000 points was found to be a good balance between having enough data to produce an accurate distribution while keeping a reasonably high time resolution. The 70% overlap allows the damage to be identified earlier than if there was no overlap; a sufficient change in structural behaviour data needs to be present in the window for the damage to be detected.
4. Fit a probability density estimate to each of the individual time window’s residuals and find the residuals’ mean. Test each window to see if the testing residuals’ mean (black dashed-dot line in Figure 17) for that window is outside of the threshold values (two green lines in Figure 17).
5. To identify the minimum frequency shift that can reliably be detected, the frequency shift was incrementally increased in the raw frequency data (usually by 0.01 Hz) until both the following conditions have been satisfied:
  (a) The first window that detects the shift includes the point at which the shift was implemented. This ensures that fully healthy windows of data never indicate damage.
  (b) All windows after the first detection also detect the shift. This ensures that after the damage is detected, all subsequent windows detect damage.

### 7.4. Testing Data Models to Determine the Identifiable Shift in Frequency

The final step in validating the MID process is identifying the level of natural frequency shift that can be detected by each of the data models. This artificial frequency shift is representative of a change in the structural behaviour of the bridge. The process described in Section 7.3 was used for all of the data models to determine this frequency shift.

**Figure 18:**
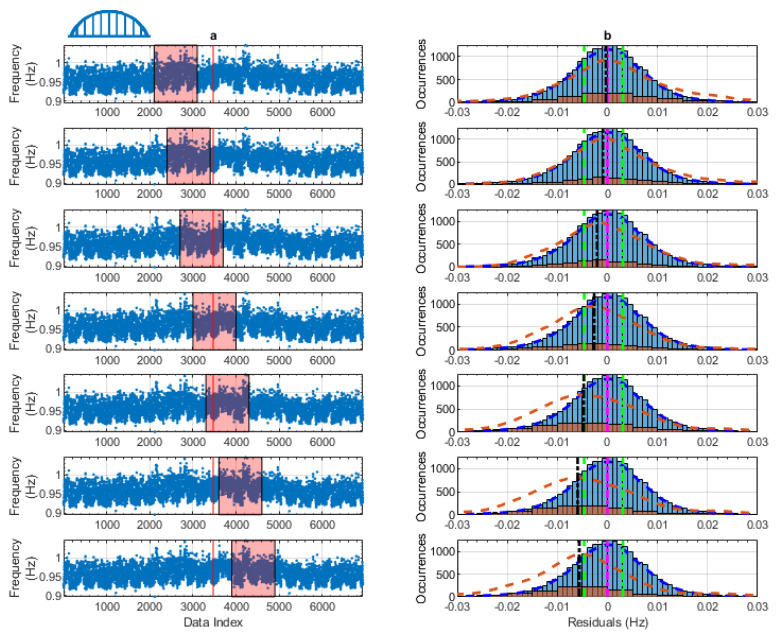
Process for identifying the frequency shift in the Bridge 1, mode 1 column. (**a**) Sections of the testing data tested for the frequency shift column. (**b**) Distributions of training data and tested data in the red box.

Figure 18 shows the process described on a sample of the testing residuals from the first mode of Bridge 1. The figure has been split into two columns, labelled ‘a’ and ‘b’. The data in column ‘a’ in the figure is the natural frequency measurements from the testing data (blue dots) after a frequency shift of −0.005 Hz is applied at the halfway point of the testing data (red line). The halfway point in the testing residual is indicated with a solid red line in column (a) in Figure 18. The plots in column ‘a’ show how the testing data were split with 1000 points of data within the red boxes. Column ‘b’ in Figure 18 shows the data contained in each of the corresponding red boxes. These plots are in the same format as Figure 17. Testing of each of the data windows is undertaken in the same manner described above; the frequency shift is detected if the testing residuals’ mean (black dashed-dot line) is outside the threshold values (two green lines). In the figure, it can be observed that the first four windows (top four rows) do not detect a frequency shift. This is expected, as these four windows contain data from before the frequency shift was introduced. In the last three windows, the mean of the testing data is beyond the threshold values, indicating that the frequency shift has been detected. The two conditions stated in Section 7.3 have been met, meaning that this data model can detect a frequency shift of 0.005 Hz. For presentation purposes, only the middle windows are shown (seven windows at the start have been omitted and six at the end).

Using the method described, the minimum frequency shift that each of the 15 data models could reliably detect was identified. The magnitudes of the frequency shifts that could be detected were identified, and they are shown in Table 4, along with the mean frequency and the frequency range. For Bridge 1, mode 4, the discrepancies that were identified in the testing data and discussed in Section 7.2 mean that the detectable magnitude of the frequency shift does not hold much value. In Table 4, the following observations can be made:

- The lowest detectable shifts for Bridges 1–4 are 0.005 Hz, 0.009 Hz, 0.01 Hz, and 0.01 Hz, respectively (indicated in bold in the table).
- Overall, the lowest and highest detectable frequency shifts that can be identified are 0.005 Hz and 0.06 Hz, respectively (shown underlined in the table).
- The range of the mode tends to influence the magnitude of the frequency shift that can be detected. Smaller ranges tend to facilitate the detection of small frequency shifts. However, there are exceptions; the bridge and mode with the largest range (Bridge 3, mode 3) can detect a frequency shift of 0.01 Hz, which is on the lower end of what can be detected by the data models.
- The mean of the natural frequency does not seem to significantly influence the magnitude of the detectable frequency shift.

The average detectable frequency shift of all 15 data models was 0.021 Hz. To put this magnitude into context, Table 5 summarises the frequency shifts caused by damage found in other studies. The frequency shifts range from minor damage, causing a 0.003 Hz shift, to a loss of 73% of the pre-stressing load, causing a 1.79 Hz shift. The information contained in Table 5 gives an indication of the size of the frequency shift that needs to be identifiable by the data model if damage is to be detected. While the studies are all different, in Table 5, it appears that low-severity damage generates frequency shifts of the order of 0.01 Hz, with larger damage resulting in shifts of the order of 0.2 Hz. One of the best benchmark studies is [29] and their work on the Z24 bridge. Here, the authors caused significant damage to the bridge by simulating a pier settlement by up to 80 mm. With the damage to the pier, the natural frequency of the bridge changed by 0.12 Hz of one mode.

Comparing the detectable frequency shifts in Table 4 to the frequency shifts due to damage reported by others (Table 5), the data models would be able to detect a large proportion of the damage implemented in the reviewed studies. While this is not definitive proof that the data models could detect damage, it shows that the frequency shift they can detect is the same order of magnitude as that caused by damage. It should be noted here that different types of damage or damage on long-span bridges that do not affect the global stiffness of the structure will most likely not be able to be detected by MID.

## 8. Conclusions

In this paper, we sought to address the following research question: Can the Minimal Information Data-modelling (MID) method be refined to be robust and consistent across a range of bridge structures? Our main result indicates that MID demonstrated the ability to accurately predict the dynamic behaviour across the four bridges tested and successfully identified frequency shifts comparable to other studies using fewer sensors and data inputs.

The accuracy and reliability of the MID method, as demonstrated by our findings, hold significant implications for bridge management practices. Despite using minimal data inputs, MID’s performance remains comparable to other more resource-intensive methods, rendering it particularly appealing to bridge managers seeking efficient yet accurate assessment tools for their infrastructure assets.

Moreover, the use of only two sensors in the MID method presents a practical advantage, suggesting the feasibility of deploying sensors across an entire bridge network. This scalability further enhances the potential applicability and cost-effectiveness of the MID method in real-world bridge monitoring scenarios.

To give context to the cost–benefit analysis of the MID approach compared to traditional SHM methods, several key points must be considered. Firstly, the lack of detailed cost reporting in existing SHM studies makes it difficult to perform a direct cost comparison. SHM encompasses a variety of methods and technologies, each with different costs. This diversity complicates the establishment of a standard baseline for comparison. In our study, the hardware costs of the MID system are minimal due to the use of low-cost sensors and components, positioning it as a highly economical option. Our primary objective was to demonstrate the feasibility and potential advantages of the MID system, highlighting that the hardware costs are among the lowest for effective SHM.

However, the equipment used in this study was standalone sensors that stored data locally. As such, site visits were required so that the batteries could be replaced and the data downloaded. Using these types of sensors across an entire bridge network would not be practical, as undertaking site visits to collect the data is not a scalable approach. To eliminate the need for site visits to collect data, a gateway and solar panel could be included in the SHM system to transfer the data to a central server. However, the transfer of raw acceleration data would be expensive in terms of both power usage and bandwidth. An alternative (or complementary) approach would be to undertake the frequency extraction locally within the SHM system and only transfer the frequency and temperature data. Using the scanning rates proposed in this study would reduce the data transfer requirements from 128 readings per second to around 10 readings (predictor + frequency) every 30 min. For this to be feasible, further investigation would be required to automate the processing of the acceleration data. This approach would also risk losing the raw data and, therefore, the potential for data validation.

The MID method, as presented in this work, falls within Level 1 of Rytter’s classifications, focusing on detecting the presence of damage to the structure. While we do not foresee MID advancing beyond the detection of damage presence, a damage detection system installed across an entire bridge network would be highly beneficial. The ability to identify damage at an early stage can significantly enhance the maintenance of bridges by enabling timely interventions. However, several limitations are associated with the MID method. MID relies on data from a limited number of sensors, making it susceptible to failures if a single sensor malfunctions. Additionally, on long-span bridges, the sparse sensor network may struggle to detect damage reliably. Despite these limitations, the MID method offers a valuable, low-cost solution for initial damage detection across a bridge network, providing an economical and effective approach to SHM. In conclusion, our research underscores the potential of the refined MID method as a robust and efficient approach to bridge management. Its accuracy, scalability, and comparative performance position it as a valuable tool for bridge managers aiming to optimise maintenance efforts across their bridge networks.

## Figures and Tables

**Table 1:** Modal analysis results for all bridges in this study.

	Bridge 1	Bridge 2	Bridge 3	Bridge 4
	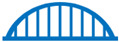	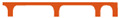	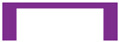	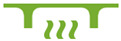
Mode 1	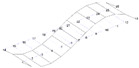	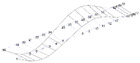	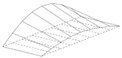	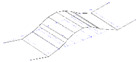
	ω_1_: 0.94 Hz ζ_1_: 3.2%	ω_1_: 2.72 Hz ζ_1_: 2.8%	ω_1_: 4.27 Hz ζ_1_: 2.1%	ω_1_: 5.54 Hz ζ_1_: 1.8%
Mode 2	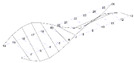	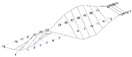	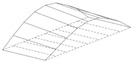	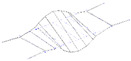
	ω_2_: 1.62 Hz ζ_2_: 2.7%	ω_2_: 3.68 Hz ζ_2_: 1.5%	ω_2_: 7.23 Hz ζ_2_: 2.1%	ω_2_: 7.54 Hz ζ_2_: 2.0%
Mode 3	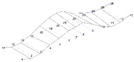	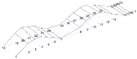	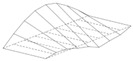	
	ω_3_: 1.74 Hz ζ_3_: 1.1%	ω_3_: 4.15 Hz ζ_3_: 1.7%	ω_3_: 9.70 Hz ζ_3_: 3.1 %	
Mode 4	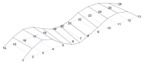	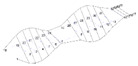		
	ω_4_: 2.75 Hz ζ_4_: 1.7%	ω_4_: 4.84 Hz ζ_4_: 1.2%		
Mode 5	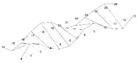	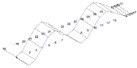		
	ω_5_: 5.08 Hz ζ_5_: 1.5%	ω_5_: 9.32 Hz ζ_5_: 1.7%		

**Table 2:** Statistics of the identified natural frequencies. (blue 17–13%, green 13–9%, red 9–4%).

Bridge Reference		Bridge 1	Bridge 2	Bridge 3	Bridge 4
Bridge Icon		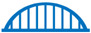	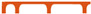	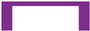	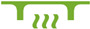
Mode 1	Average Frequency (Hz)	0.95	2.58	4.44	5.67
	Relative Variation (%)	16.41	14.64	7.65	12.24
Mode 2	Average Frequency (Hz)	1.62	3.71	7.35	7.81
	Relative Variation (%)	8.55	4.87	13.17	16.37
Mode 3	Average Frequency (Hz)	1.76	4.05	9.99	
	Relative Variation (%)	5.45	9.47	16.22	
Mode 4	Average Frequency (Hz)	2.76	4.86		
	Relative Variation (%)	8.63	5.44		
Mode 5	Average Frequency (Hz)	5.19	9.33		
	Relative Variation (%)	7.21	5.13		

**Table 3:** Histograms showing training (blue) and testing (orange) residuals for each of the 15 data models.

	Bridge 1	Bridge 2	Bridge 3	Bridge 4
	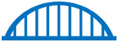	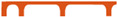	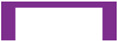	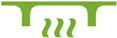
Mode 1	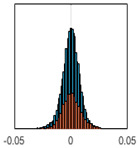	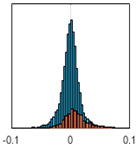	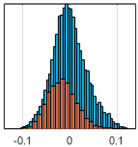	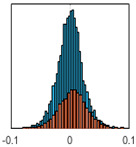
Mode 2	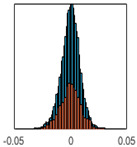	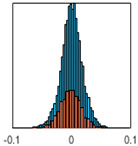	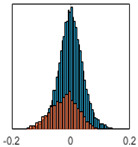	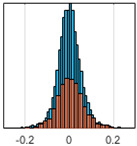
Mode 3	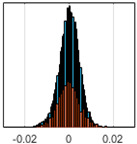	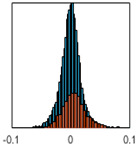	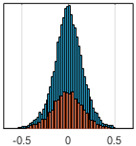	
Mode 4	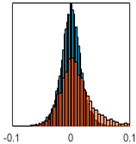	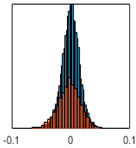		
Mode 5	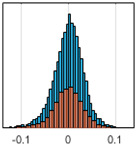	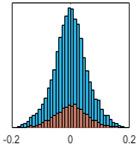		

**Table 4:** Comparison of data model results. lowest detectable shifts for each bridge shown in bold. Lowest and highest detectable frequency underlined.

		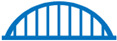	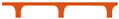	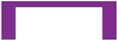	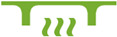
		Bridge 1	Bridge 2	Bridge 3	Bridge 4
Mode 1	Mean (Hz)	0.95	2.58	4.44	5.67
	Range (Hz)	0.157	0.378	0.347	0.635
	Detect (Hz)	**0.005**	0.02	0.06	**0.01**
Mode 2	Mean (Hz)	1.62	3.71	7.35	7.81
	Range (Hz)	0.143	0.181	0.66	1.064
	Detect (Hz)	0.007	0.03	0.03	**0.01**
Mode 3	Mean (Hz)	1.76	4.05	9.99	
	Range (Hz)	0.095	0.383	1.62	
	Detect (Hz)	0.008	**0.009**	**0.01**	
Mode 4	Mean (Hz)	2.76	4.86		
	Range (Hz)	0.238	0.257		
	Detect (Hz)	See Section 7.2	0.02		
Mode 5	Mean (Hz)	5.19	9.33		
	Range (Hz)	0.373	0.479		
	Detect (Hz)	0.03	0.05		

**Table 5:** Frequency shift caused by damage found in other studies.

Reference	Test Structure	Damage	Frequency Shift Caused by Damage
[29]	Z24 bridge—post-tensioned concrete box girder bridge	Pier settlement (20 mm and 80 mm)	Modes (4) showed a change between 0.01 Hz and 0.12 Hz
[30]	Laboratory test of pre-stressed RC beam (6 m)	Releasing the pre-stressed tendon from 17% to 73%	Modes (4) showed a change between 0.13 Hz and 1.79 Hz
[31]	Numerical analysis of the Champangshiehl Bridge	A series of damages were introduced relating to pre-stressed tendons	Modes (2) showed a change between 0.03 Hz and 0.17 Hz
[32]	I-40 Bridge—concrete deck supported by two steel plate girders	Cutting a beam on the bridge by varying amounts	Modes (6) showed a change between 0.01 Hz and 0.18 Hz
[20]	FE model of a 110 m arch footbridge	Damage was simulated by changing the spring stiffness between the piles and the bridge	Modes (4) showed a change between 0.00 Hz and 0.097 Hz
[21]	FE Model of a stress ribbon footbridge	Damage was represented by different spring elements’ stiffness being modelled	Modes (12) showed a change between 0.003 Hz and 0.961 Hz

## Data Availability

Some models or codes that support the findings of this study are available from the corresponding author upon reasonable request.
